# Predictive Role of NLR, dNLR, PLR, NLPR, and Other Laboratory Markers in Diagnosing SIRS in Premature Newborns

**DOI:** 10.3390/clinpract14030084

**Published:** 2024-06-06

**Authors:** Manuela Pantea, Daniela Iacob, Claudia Ioana Bortea, Ileana Enatescu, Vlad Barbos, Mihaela Prodan, Raluca Tudor, Gabriel Veniamin Cozma

**Affiliations:** 1 Department of Neonatology, “Victor Babes” University of Medicine and Pharmacy Timisoara, 300041 Timisoara, Romania; manuela.pantea@umft.ro (M.P.); iacob.daniela@umft.ro (D.I.); bortea.ioana@umft.ro (C.I.B.); enatescu.ileana@umft.ro (I.E.); 2 Doctoral School, “Victor Babes” University of Medicine and Pharmacy Timisoara, 300041 Timisoara, Romania; vlad.barbos@umft.ro (V.B.); mihaela.prodan@umft.ro (M.P.); 3 Second Discipline of Neurology, “Victor Babes” University of Medicine and Pharmacy Timisoara, 300041 Timisoara, Romania; 4 Department of Surgical Semiology I and Thoracic Surgery, “Victor Babes” University of Medicine and Pharmacy of Timisoara, 300041 Timisoara, Romania; gabriel.cozma@umft.ro

**Keywords:** prematurity, preterm birth, inflammatory markers, risk analysis, neonatology

## Abstract

Background: Premature newborns are at a significant risk for Systemic Inflammatory Response Syndrome SIRS, a condition associated with high morbidity and mortality. This study aimed to evaluate the predictive and diagnostic capability of laboratory markers like Neutrophil to Lymphocyte Ratio (NLR), derived Neutrophil to Lymphocyte Ratio (dNLR), Platelet-to-Lymphocyte Ratio (PLR), and Neutrophil-to-Lymphocyte-to-Platelet Ratio (NLPR) in diagnosing SIRS in premature newborns. Methods: Premature newborns with and without SIRS were evaluated in a prospective design during a one-year period. Among 136 newborns, early and 72 h post-birth analyses were performed. Results: At 24 h, NLR’s cutoff value was 8.69, yielding sensitivity and specificity rates of 52.77% and 83.47% (*p* = 0.0429), respectively. The dNLR showed a cutoff of 5.61, with corresponding rates of 63.27% and 84.15% (*p* = 0.0011), PLR had a cutoff of 408.75, with rates of 51.89% and 80.22% (*p* = 0.1026), and NLPR displayed a cutoff of 0.24, with rates of 75.85% and 86.70% (*p* = 0.0002). At 72 h, notable sensitivity and specificity improvements were observed, particularly with NLPR having a cutoff of 0.17, showing sensitivity of 77.74% and specificity of 95.18% (*p* < 0.0001). NLR above the cutoff indicated a 33% increase in SIRS risk, with a hazard ratio (HR)of 1.33. The dNLR was associated with a twofold increase in risk (HR 2.04). NLPR demonstrated a significant, over threefold increase in SIRS risk (HR 3.56), underscoring its strong predictive and diagnostic value for SIRS development. Conclusion: Integrating these findings into clinical practice could enhance neonatal care by facilitating the early identification and management of SIRS, potentially improving outcomes for this vulnerable population.

## 1. Introduction

Preterm birth, defined as delivery before 37 weeks of gestation, is a leading cause of neonatal morbidity and mortality worldwide [1,2,3,4]. The prematurity status places these infants at a significant disadvantage, primarily due to the underdevelopment of their organ systems, including the immune system. This immaturity renders them highly susceptible to infections and systemic inflammatory conditions, of which the Systemic Inflammatory Response Syndrome (SIRS) is particularly concerning, triggered by a multitude of neonatal and maternal factors [5,6,7,8]. SIRS in premature newborns can lead to a cascade of complications, further endangering their already fragile health [9]. Given these risks, the medical community has an ongoing interest in identifying and mitigating the factors contributing to the development of SIRS and associated complications that can have long-term consequences and complications in adult life [10,11,12,13,14].

Premature infants are at a heightened risk of developing SIRS due to several factors, such as their underdeveloped immune system, which is less capable of developing an appropriate response to pathogens or other inflammatory stimuli, making them more susceptible to infections that can trigger SIRS [15]. Moreover, the procedures and interventions that premature newborns or their mothers often undergo, such as mechanical ventilation and central venous catheterization, can also increase the risk of systemic infection and inflammation [16,17,18]. The development of SIRS in these infants is particularly troubling, as it can rapidly progress to severe complications like sepsis, multi-organ dysfunction syndrome, and even death [19].

Traditional clinical signs and laboratory markers of inflammation and infection can be nonspecific and late-appearing in this population, leading to delays in diagnosis and treatment [20]. In this context, hematological biomarkers and scores, such as the Neutrophil-to-Lymphocyte Ratio (NLR), derived Neutrophil-to-Lymphocyte Ratio (dNLR), Platelet-to-Lymphocyte Ratio (PLR), and Neutrophil-to-Lymphocyte-to-Platelet Ratio (NLPR), have emerged as promising candidates, among other biomarkers [21,22,23,24]. These biomarkers, derived from routine complete blood counts, offer a non-invasive, cost-effective, and rapidly accessible means to evaluate systemic inflammation and infection risk. Preliminary studies in adult and pediatric populations have shown their potential to predict the severity and outcome of various inflammatory conditions [25]. However, their utility in the premature neonatal population is still a question of debate.

The objectives of this study are, therefore, to systematically assess the predictive potential of NLR, dNLR, PLR, NLPR, and other laboratory markers in diagnosing the occurrence of SIRS in premature newborns. By exploring these relationships, the study aims to identify reliable, simple, and accessible biomarkers that could enhance the early detection of SIRS and development of neonatal sepsis.

## 2. Materials and Methods

### 2.1. Study Design and Ethical Considerations

This prospective cohort study was conducted over a period of one year, spanning from January 2023 to January 2024. The research was centered within the Neonatal Unit at the Pius Brinzeu Emergency Clinical Hospital in Timisoara, focusing on premature neonates that developed SIRS, in comparison to a control group of preterm newborns without SIRS.

The study adhered strictly to the ethical standards set by the institutional research committee and was in alignment with the principles of the 1964 Helsinki Declaration and its subsequent amendments concerning ethical standards in medical research. The research protocol received thorough review and approval from the Ethical Committee for Scientific Research at the Victor Babes University of Medicine and Pharmacy Timisoara. The approval, granted on 29 November 2022, was documented under approval number 349. Prior to inclusion in the study, informed consent was secured from the parents or legal guardians of all neonatal participants, ensuring understanding and voluntary participation in the research.

### 2.2. Inclusion and Exclusion Criteria

The inclusion criteria for this study comprised: (1) preterm newborns with a gestational age (GA) including extremely preterm (less than 28 weeks), very preterm (28–31 weeks), and moderate to late preterm (32–36 weeks) infants; (2) newborns that had measurements for inflammatory markers and laboratory parameters required to determine the NLR, dNLR, PLR, and NLPR. Conversely, the exclusion criteria were distinctly defined to maintain the study’s focus and integrity: (1) the presence of severe congenital anomalies, particularly those affecting the cardiac, pulmonary, or central nervous systems, due to their potential independent impact laboratory studies; (2) neonates diagnosed with genetic syndromes, given the complex interplay between genetic factors and neonatal health outcomes; (3) infants who died during the neonatal period; (4) cases where informed consent for participation in the study and data collection was not obtained from the parents or legal guardians, adhering to ethical standards for human research.

SIRS in newborns is defined by the presence of more than one of the following clinical findings, according to current guidelines [18]: a core body temperature of <36 °C or >38.5 °C; a heart rate of >180 beats per minute for at least 30 min; a respiratory rate of >60 breaths per minute or the need for mechanical ventilation not due to congenital reasons; and a white blood cell count of <5000 cells/mm^3^ or >15,000 cells/mm^3^.

### 2.3. Biochemical Analysis

For the complete blood count (CBC), including the white blood cell (WBC) count, a Sysmex XN-550 automated hematology analyzer provided by Sysmex Corporation, Kobe, Japan, was employed. This involved collecting 1 mL of peripheral venous blood from each participating neonate into a test tube containing Ethylenediaminetetraacetic acid (EDTA) as an anticoagulant, which prevents blood clotting and preserves the sample integrity for accurate analysis.

The levels of C-Reactive Protein (CRP) were determined using either a Cobas Integra 400 Plus or Cobas e411 analyzer from Roche Diagnostics GmbH, Mannheim, Germany. This assay required 2 mL of peripheral venous blood, collected in tubes without anticoagulants but with a separator gel, which facilitates the separation of serum from blood cells for analysis.

For the measurement of Lactate Dehydrogenase (LDH), Creatine Kinase (CK), Aspartate Aminotransferase (AST), and Alanine Aminotransferase (ALT), the study utilized biochemical analyzers that operate on the principle of spectrophotometry. The parameters of blood gas analysis, including pH, pCO_2_ (partial pressure of carbon dioxide), and pO_2_ (partial pressure of oxygen), were determined using a blood gas analyzer.

Blood sample collections were strategically scheduled at two postnatal intervals. The initial collection (time point 1) was performed within the first hours following birth to establish baseline levels of the markers under investigation. A subsequent collection (time point 2) occurred at 72 h of postnatal life, a critical timeframe for detecting any notable changes in the biochemical markers that could indicate the development of SIRS in premature newborns.

Key to the study’s biochemical analysis were the calculations of ratios known to reflect immune response dynamics and the potential onset of SIRS in premature neonates. The NLR was calculated by dividing the absolute neutrophil count by the absolute lymphocyte count. The dNLR was determined by dividing the absolute neutrophil count by the difference between the total white blood cell count and the absolute neutrophil count. The PLR was calculated by dividing the absolute platelet count by the absolute lymphocyte count. Finally, the NLPR was calculated using a formula that integrates the counts of neutrophils, lymphocytes, and platelets into a single ratio.

### 2.4. Statistical Analysis

Data management and analysis were conducted utilizing the statistical software SPSS version 26.0 (SPSS Inc., Chicago, IL, USA). English editing was performed using ChatGPT version 3.5 (OpenAI, San Francisco, CA, USA). Continuous variables were represented as mean ± standard deviation (SD), while categorical variables were expressed in terms of frequencies and percentages. The Student’s *t*-test for comparing two means between the continuous data. The Chi-square test was utilized for the categorical variables. The best cutoff value, sensitivity, specificity, Area Under Curve (AUC), and the Receiver Operating Characteristic were calculated to determine the prediction and diagnostic value of the proposed parameters. A regression analysis adjusted for gestational age and gestational weight was performed to identify the hazard ratio of developing SIRS based on the laboratory parameters that cross the calculated cutoff values. A *p*-value threshold of less than 0.05 was set for statistical significance. All results were double-checked to ensure accuracy and reliability.

## 3. Results

The final study included 53 preterm newborns who developed SIRS and 83 preterm newborns who did not develop SIRS, as presented in Table 1. Statistical analysis revealed significant differences in gestational age between newborns with SIRS (30.74 weeks) and those without SIRS (32.28 weeks), with a *p*-value of 0.002. Similarly, gestational weight showed significant differences between the two groups, with the SIRS group having a lower mean gestational weight (1505.19 g) compared to the no SIRS group (1838.13 g), and this difference was statistically significant (*p* = 0.001).

Moreover, the distribution of APGAR scores ≤ 7 was significantly higher in the SIRS group compared to the no SIRS group, with a *p*-value of 0.031. Another notable finding was the incidence of GBS-positive cultures among the newborn mothers, which was significantly higher in the SIRS group (30.19%) compared to the no SIRS group, where there were no cases (*p*-value < 0.001). A total of 21 newborns (39.62%) had culture-positive sepsis, as presented in Table 1.

In the comparison of laboratory parameters on the first day of life between newborns with SIRS and those without, the analysis reveals distinct patterns across various biomarkers. Notably, the pH, carbon dioxide (pCO_2_), and oxygen (pO_2_) levels, along with lactate, did not show statistically significant differences between the two groups. Specifically, pH levels were slightly lower in the SIRS group (7.29) compared to the non-SIRS group (7.31), but with a *p*-value of 0.3549, suggesting no significant difference. Similarly, pCO_2_ and pO_2_ levels, along with lactate concentrations, also showed no significant difference between the groups.

In contrast, WBC and differentiation parameters such as neutrophils and lymphocytes presented significant differences. Newborns with SIRS had higher WBC counts (16.46 × 10^9^/L) compared to those without (12.73 × 10^9^/L), with a *p*-value of 0.003. Neutrophils and lymphocytes also followed this trend, with neutrophils showing a mean count higher in the SIRS group (8.76 × 10^9^/L) than in the non-SIRS group (6.56 × 10^9^/L), achieving statistical significance with a *p*-value of 0.017. Lymphocytes were also significantly higher in the SIRS group (6.88 × 10^9^/L) compared to the non-SIRS group (4.86 × 10^9^/L), with a *p*-value of 0.003.

Platelet counts and inflammatory markers further distinguished the two groups. The platelet count was notably lower in newborns with SIRS (207.92 × 10^9^/L) compared to those without (260.83 × 10^9^/L), with the difference being highly significant (*p* < 0.001). C-Reactive Protein (CRP) levels, an acute-phase reactant, were also significantly higher in the SIRS group (8.76 mg/L) compared to the non-SIRS group (4.29 mg/L), with a *p*-value of 0.008. Ratios of NLR, PLR, dNLR, and NLPR were also examined. The NLR and dNLR were significantly higher in the SIRS group, with *p*-values of 0.030 and <0.001, respectively. The NLPR also showed a significant difference, with a much higher mean in the SIRS group compared to the non-SIRS group (0.29 vs. 0.13), with a *p*-value of <0.001, as described in Table 2.

By 72 h from birth, the comparative analysis of laboratory parameters between newborns with and without SIRS continued to show significant differences. Significant disparities were observed in the NLR, dNLR, PLR, and NLPR values between the two groups, indicating their potential as biomarkers for SIRS in premature newborns. The NLR significantly increased in newborns with SIRS (4.59 ± 2.61) compared to those without (2.41 ± 4.38), with a *p*-value of 0.001.

The dNLR also showed a pronounced difference, with values notably higher in the SIRS group (2.81 ± 1.76) versus the non-SIRS group (1.37 ± 1.05), the difference being statistically significant (*p*-value < 0.001). PLR also presented a significant difference, standing at 114.33 ± 163.02 for the SIRS group compared to 71.72 ± 61.14 for the non-SIRS group, with a *p*-value of 0.033. Lastly, the NLPR ratio further delineated the groups, with the SIRS newborns showing a value of 0.27 ± 0.48 compared to 0.07 ± 0.05 in the non-SIRS group, marked by a *p*-value of 0.001, as described in Table 3.

For the laboratory parameters assessed at 24 h from birth, the Neutrophil-to-Lymphocyte Ratio (NLR) displayed a cutoff value of 8.69, with sensitivity and specificity rates of 52.77% and 83.47%, respectively (*p* = 0.043). The Derived Neutrophil-to-Lymphocyte Ratio (dNLR) exhibited a cutoff value of 5.61, with sensitivity and specificity rates of 63.27% and 84.15% respectively (*p* = 0.0011). Platelet-to-Lymphocyte Ratio (PLR) demonstrated a cutoff value of 408.75, with sensitivity and specificity rates of 51.89% and 80.22%, respectively (*p* = 0.103). Lastly, the Neutrophil, Lymphocyte, and Platelet Ratio (NLPR) presented a cutoff value of 0.24, with sensitivity and specificity rates of 75.85% and 86.70%, respectively (*p* = 0.001).

Regarding the laboratory parameters evaluated at 72 h from birth, the Neutrophil-to-Lymphocyte Ratio (NLR) displayed a cutoff value of 4.40, with sensitivity and specificity rates of 62.64% and 91.57%, respectively (*p* = 0.004). The Derived Neutrophil-to-Lymphocyte Ratio (dNLR) exhibited a cutoff value of 2.88, with sensitivity and specificity rates of 24.53% and 82.39%, respectively (*p* = 0.001). Platelet-to-Lymphocyte Ratio (PLR) demonstrated a cutoff value of 158.86, with sensitivity and specificity rates of 59.43% and 78.56%, respectively (*p* = 0.002). Lastly, the Neutrophil, Lymphocyte, and Platelet Ratio (NLPR) presented a cutoff value of 0.17, with sensitivity and specificity rates of 77.74% and 95.18%, respectively (*p* < 0.001), as presented in Table 4.

**Figure 1:**
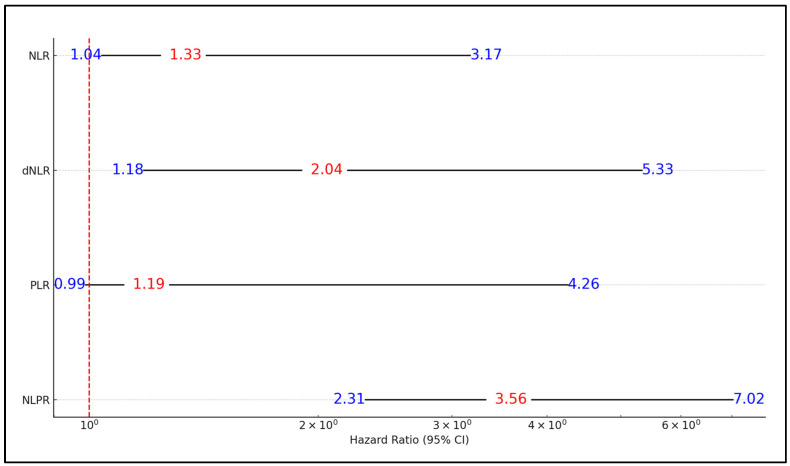
Forest plot for the risk of SIRS development based on the best cutoff values.

In the regression analysis for SIRS development, factors above the best cutoff values exhibited varying hazard ratios (HR) and significance levels. Specifically, the Neutrophil-to-Lymphocyte Ratio (NLR) displayed a hazard ratio of 1.33 (95% CI: 1.04–3.17) with a significant *p*-value of 0.004, indicating a positive association with SIRS development. The Derived Neutrophil-to-Lymphocyte Ratio (dNLR) showed a higher hazard ratio of 2.04 (95% CI: 1.18–5.33) with a highly significant *p*-value of 0.001, suggesting a stronger association with SIRS. Platelet-to-Lymphocyte Ratio (PLR) exhibited a hazard ratio of 1.19 (95% CI: 0.99–4.26) with a borderline significant *p*-value of 0.051, indicating a possible but less robust association with SIRS. Conversely, the Neutrophil, Lymphocyte, and Platelet Ratio (NLPR) demonstrated the highest hazard ratio of 3.56 (95% CI: 2.31–7.02) with an extremely significant *p*-value of <0.001, highlighting a substantial positive association with SIRS development, as presented in Table 5 and Figure 1.

## 4. Discussion

### 4.1. Literature Findings

The study’s analysis unveils a nuanced understanding of laboratory markers in predicting and diagnosing SIRS among premature newborns. Initial findings suggest that conventional markers such as pH, pCO_2_, pO_2_, and lactate levels, despite their clinical significance in neonatal care, do not present a significant difference between newborns who developed SIRS and those who did not. Further emphasizing the diagnostic landscape of neonatal SIRS, the study delineates the significant role of platelet counts and inflammatory markers like CRP, alongside ratios such as NLR, dNLR, and NLPR. The pronounced differences in these parameters in newborns who developed SIRS not only confirm their relevance in diagnosing SIRS but also hint at their underlying mechanisms in neonatal inflammation. Particularly, the inverse relationship between platelet count and SIRS prevalence, coupled with elevated CRP levels, suggests a complex interplay of hemostatic and inflammatory processes in neonatal SIRS. These findings, especially the significant distinctions in NLR, dNLR, and NLPR ratios, reinforce the potential of these markers in refining the diagnostic criteria for SIRS in premature newborns.

As reported by existing literature, the regression analysis performed in this study further cements the diagnostic value of specific ratios above their best cutoff values, elucidating their varying degrees of association with SIRS development. The NLR and dNLR, along with the NLPR, present a quantifiable risk of SIRS, each offering a unique lens through which the inflammatory process can be assessed and managed, and in accordance with other studies. The studies by Domnicu et al. [26] and Li et al. [27] both affirm the pivotal role of the NLR in diagnosing and assessing the risk of sepsis in vulnerable pediatric populations, although with different focal groups and outcomes. Domnicu et al. [26] concentrate on malnourished infants, finding NLR alongside procalcitonin as the most accurate discriminators for sepsis, with NLR showing a significant, gradual increase from sepsis to septic shock and correlating strongly with adverse outcomes like prolonged ICU stay and higher mortality. Dong et al. [27], surveying a broader cohort of 1480 neonates, identify NLR as an independent risk factor for neonatal sepsis, noting a significant rise in sepsis risk with NLR elevations, specifically, the risk of sepsis climbing from 41.6% at an NLR below 0.91 to 66.2% when NLR exceeded 1.88, with an optimal NLR cutoff of 1.62 for sepsis prediction (AUC = 0.63). These studies fall in accordance with our findings and collectively underscore the diagnostic and prognostic utility of NLR in a pediatric setting.

Sumitro et al.’s cross-sectional study [28] identified NLR as a viable, cost-effective alternative marker for neonatal sepsis in developing countries, showcasing a median NLR value of 3.63 in neonates with sepsis and establishing an NLR cut-off of ≥2.12 as significantly associated with positive blood culture results (relative risk = 1.867, *p* = 0.011), especially when combined with a CRP level of ≥2.70 mg/dL. Conversely, Xin et al. [29] aggregate data across 14 studies encompassing 1499 newborns, revealing a broad NLR cut-off range from 0.1 to 9.4 for sepsis diagnosis. Their findings highlight an NLR with a pooled sensitivity of 0.74, specificity of 0.88, and an AUC of 0.87 for general neonatal sepsis diagnosis, with even higher accuracy in early-onset neonatal sepsis (AUC = 0.97), significantly higher than identified in our study.

Similarly, Thakur et al. [30] and Rana et al. [31] provided complementary insights into the early detection of neonatal sepsis, albeit through distinct methodological lenses. Thakur et al. challenged the conventional reliance on SIRS criteria by introducing two predictive models, Model A and Model B, which demonstrate superior diagnostic accuracy with significantly better sensitivity (29.17% and 31.25%), specificity (97.82% and 97.30%), and likelihood ratios (PLR: 13.36 and 11.56; NLR: 0.72 and 0.71) compared to traditional SIRS, validated further through an accessible android application for clinical use. On the other hand, Rana et al. underscored the value of the NLR in a retrospective study, revealing it as significant, though having only modest power (AUC = 0.569) as a predictor of neonatal sepsis with median NLR levels markedly higher in septic neonates, offering a cost-effective and straightforward diagnostic marker.

Bai et al.’s meta-analysis [32] encompassed 2610 participants, highlighting the NLR and PLR with sensitivities of 0.76 and 0.82, specificities of 0.82 and 0.80, and AUC values of 0.86 and 0.87, respectively, indicating both ratios’ high diagnostic accuracy for neonatal sepsis. Zhang et al. [33] expanded the scope to include immature-to-total (ITR) and immature-to-mature neutrophil ratios (IMR), with their study showing ITR and IMR providing summary sensitivities of 0.74 each and higher specificities of 0.83 and 0.89, respectively, compared to NLR’s lower specificity of 0.69. These findings underscore the potential of these hematological ratios in enhancing NS diagnosis, though Zhang et al.’s caution regarding NLR’s precision suggests a need for integrating multiple diagnostic indicators to improve reliability and outcomes in neonatal care.

The clinical utility of NLR, dNLR, NLPR, and PLR in the context of neonatal care, especially in the early diagnosis and risk assessment of SIRS in premature newborns, is substantial. These markers, by reflecting the underlying inflammation and immune response, offer clinicians a set of powerful, non-invasive tools for early detection and management strategies for SIRS, potentially reducing the risk of progression to more severe conditions. Their sensitivity and specificity, as reported in various studies, suggest that these ratios can serve as effective adjuncts to traditional diagnostic methods, providing valuable insights into the severity of the condition and guiding therapeutic decisions. Moreover, the cost-effectiveness and ease of calculation from routine blood tests make NLR, dNLR, NLPR, and PLR particularly appealing for use in diverse clinical settings, including resource-limited environments.

### 4.2. Limitations and Future Perspectives

The study, while providing valuable insights into the predictive role of NLR, dNLR, PLR, NLPR, and other laboratory markers for diagnosing SIRS in premature newborns, faces several limitations that warrant attention. The observational cohort design, though comprehensive, restricts the ability to infer causality between the identified biomarkers and the onset of SIRS, limiting findings to associations. The study’s reliance on a single neonatal unit and the small sample size may also affect the generalizability of results across diverse neonatal care settings, potentially limiting applicability to wider populations due to regional variations in neonatal care practices and patient demographics. Furthermore, the significant heterogeneity identified among the laboratory parameters, especially in their diagnostic values across different time points, suggests variability that could affect diagnostic precision. Considering the small sample size collected from a single-center neonatology unit, the prediction capability for blood culture-positive sepsis was not possible. Therefore, future studies with a bigger sample size could attempt to validate these scores in diagnosing SIRS and blood culture-positive sepsis.

## 5. Conclusions

The findings challenge the reliance on traditional biomarkers, instead highlighting the diagnostic and diagnostic superiority of leukocyte profiles, platelet counts, and specific ratios such as NLR, dNLR, and NLPR. The associations between these laboratory markers and SIRS underscore their clinical utility, considering the availability of such laboratory data and the ease of calculating these scores. Nevertheless, there is a need for further research to validate these findings in larger cohorts of patients and explore their clinical applicability, with the ultimate goal of improving neonatal outcomes.

## Figures and Tables

**Table 1:** Comparison of background characteristics between newborns with and without SIRS.

Variables	SIRS (*n* = 53)	No SIRS (*n* = 83)	*p*-Value
Gestational age (mean ± SD)	30.74 ± 2.88	32.28 ± 2.65	0.002
Gestational age, n (%)			0.005
Extremely preterm (<28 weeks)	9 (16.98%)	5 (6.02%)	
Very preterm (28–31 weeks)	24 (45.28%)	24 (28.92%)	
Moderate to late preterm (32–36 weeks)	20 (37.74%)	54 (65.06%)	
Gestational weight (mean ± SD)	1505.19 ± 580.10	1838.13 ± 544.84	0.001
Gestational weight, n(%)			0.045
Extremely low (<1000 g)	12 (22.64%)	7 (8.43%)	
Very low (1000–1499 g)	15 (28.30%)	17 (20.48%)	
Low (1500–2499 g)	23 (43.40%)	53 (63.86%)	
Normal (>2500 g)	3 (5.66%)	6 (7.23%)	
Gender, n (%)			0.327
Male	32 (60.38%)	43 (51.81%)	
Female	21 (39.62%)	40 (48.19%)	
APGAR (mean ± SD)	6.34 ± 1.70	6.54 ± 1.83	0.524
APGAR, n (%)			0.031
≤7	19 (35.85%)	16 (19.28%)	
>7	34 (64.15%)	67 (80.72%)	
Newborn with GBS-positive mother	16 (30.19%)	0 (0.00%)	<0.001
Culture-positive sepsis	21 (39.62%)	-	-

SD—Standard Deviation; GBS—Group B Streptococcus; APGAR—Appearance Pulse Grimace Activity and Respiration; SIRS—Systemic Inflammatory Response Syndrome.

**Table 2:** Comparison of laboratory parameters in the first day of life between newborns with and without SIRS.

Variables (Mean ± SD)	SIRS (*n* = 53)	No SIRS (*n* = 83)	*p*-Value
pH	7.29 ± 0.14	7.31 ± 0.11	0.355
pCO_2_	45.58 ± 14.88 mmHg	44.08 ± 11.82 mmHg	0.516
pO_2_	24.30 ± 16.59 mmHg	23.13 ± 13.02 mmHg	0.647
Lactate	3.38 ± 2.30 mmol/L	2.87 ± 2.16 mmol/L	0.193
WBC	16.46 ± 10.37 × 10^9^/L	12.73 ± 3.09 × 10^9^/L	0.003
Neutrophils	8.76 ± 7.32 × 10^9^/L	6.56 ± 3.07 × 10^9^/L	0.017
Lymphocytes	6.88 ± 5.51 × 10^9^/L	4.86 ± 2.06 × 10^9^/L	0.003
Platelets	207.92 ± 73.32 × 10^9^/L	260.83 ± 70.60 × 10^9^/L	<0.001
CRP	8.76 ± 12.99 mg/L	4.29 ± 6.01 mg/L	0.008
LDH	816.08 ± 1714.42 U/L	577.07 ± 263.08 U/L	0.214
CK	290.45 ± 179.75 U/L	297.87 ± 182.96 U/L	0.817
AST	58.36 ± 32.21 U/L	62.31 ± 34.09 U/L	0.502
ALT	11.36 ± 8.01 U/L	12.87 ± 7.85 U/L	0.279
NLR	3.01 ± 4.63	1.81 ± 1.54	0.031
dNLR	2.15 ± 1.27	1.31 ± 0.91	<0.001
PLR	73.46 ± 47.85	56.16 ± 63.93	0.093
NLPR	0.29 ± 0.30	0.13 ± 0.10	<0.001

SD—Standard Deviation; SIRS—Systemic Inflammatory Response Syndrome; NLR—Neutrophil-to-Lymphocyte Ratio; dNLR—Derived Neutrophil-to-Lymphocyte Ratio; PLR—Platelet-to-Lymphocyte Ratio; NLPR—Neutrophil, Lymphocyte, and Platelet Ratio; pH—(Normal Range: 7.35–7.45); pCO_2_—Partial Pressure of Carbon Dioxide (Normal Range: 35–45 mmHg); pO_2_—Partial Pressure of Oxygen (Normal Range: 50–70 mmHg); WBC—White Blood Cells (Normal Range: 5.0–10.0 × 10^9^/L); CRP—C-Reactive Protein (Normal Range: <10 mg/L); LDH—Lactate Dehydrogenase (Normal Range: 135–225 U/L); CK—Creatine Kinase (Normal Range: 52–336 U/L); AST—Aspartate Aminotransferase (Normal Range: 0–40 U/L); ALT—Alanine Aminotransferase (Normal Range: 0–40 U/L).

**Table 3:** Comparison of laboratory parameters at 72 h from birth between newborns with and without SIRS.

Variables (Mean ± SD)	SIRS (*n* = 53)	No SIRS (*n* = 83)	*p*-Value
WBC	15.23 ± 11.33 × 10^9^/L	10.01 ± 3.50 × 10^9^/L	0.001
Neutrophils	9.21 ± 8.90 × 10^9^/L	5.35 ± 3.03 × 10^9^/L	0.001
Lymphocytes	4.54 ± 4.16 × 10^9^/L	3.40 ± 1.50 × 10^9^/L	0.024
Platelets	236.69 ± 106.54 × 10^9^/L	281.40 ± 96.68 × 10^9^/L	0.013
CRP	9.96 ± 13.60 mg/L	6.76 ± 9.27 mg/L	0.105
LDH	822.11 ± 1170.04 U/L	590.78 ± 228.71 U/L	0.082
CK	301.25 ± 335.09 U/L	271.19 ± 177.30 U/L	0.496
AST	80.47 ± 190.58 U/L	56.61 ± 29.30 U/L	0.264
ALT	18.42 ± 22.56 U/L	15.73 ± 12.34 U/L	0.371
NLR	4.59 ± 2.61	2.41 ± 4.38	0.001
dNLR	2.81 ± 1.76	1.37 ± 1.05	<0.001
PLR	114.33 ± 163.02	71.72 ± 61.14	0.033
NLPR	0.27 ± 0.48	0.07 ± 0.05	0.001

SD—Standard Deviation; SIRS—Systemic Inflammatory Response Syndrome; NLR—Neutrophil-to-Lymphocyte Ratio; dNLR—Derived Neutrophil-to-Lymphocyte Ratio; PLR—Platelet-to-Lymphocyte Ratio; NLPR—Neutrophil, Lymphocyte, and Platelet Ratio; WBC—White Blood Cells (Normal Range: 5.0–10.0 × 10^9^/L); CRP—C-Reactive Protein (Normal Range: <10 mg/L); LDH—Lactate Dehydrogenase (Normal Range: 135–225 U/L); CK—Creatine Kinase (Normal Range: 52–336 U/L); AST—Aspartate Aminotransferase (Normal Range: 0–40 U/L); ALT—Alanine Aminotransferase (Normal Range: 0–40 U/L).

**Table 4:** Best cutoff values for diagnosing SIRS in newborns.

Laboratory Parameter	Timeframe	Best Cutoff Value	Sensitivity	Specificity	AUC	*p*-Value
NLR	24 h	8.69	52.77%	83.47%	0.522	0.043
dNLR	24 h	5.61	63.27%	84.15%	0.624	0.001
PLR	24 h	408.75	51.89%	80.22%	0.501	0.103
NLPR	24 h	0.24	75.85%	86.70%	0.634	0.001
NLR	72 h	4.40	62.64%	91.57%	0.616	0.004
dNLR	72 h	2.88	24.53%	82.39%	0.621	0.001
PLR	72 h	158.86	59.43%	78.56%	0.594	0.002
NLPR	72 h	0.17	77.74%	95.18%	0.692	<0.001

SIRS—Systemic Inflammatory Response Syndrome; NLR—Neutrophil-to-Lymphocyte Ratio; dNLR—Derived Neutrophil-to-Lymphocyte Ratio; PLR—Platelet-to-Lymphocyte Ratio; NLPR—Neutrophil, Lymphocyte, and Platelet Ratio.

**Table 5:** Regression analysis for SIRS development.

Factors above the Best Cutoff	Hazard Ratio	95% CI	*p*-Value
NLR	1.33	1.04–3.17	0.004
dNLR	2.04	1.18–5.33	0.001
PLR	1.19	0.99–4.26	0.051
NLPR	3.56	2.31–7.02	<0.001

SIRS—Systemic Inflammatory Response Syndrome; NLR—Neutrophil-to-Lymphocyte Ratio; dNLR—Derived Neutrophil-to-Lymphocyte Ratio; PLR—Platelet-to-Lymphocyte Ratio; NLPR—Neutrophil, Lymphocyte, and Platelet Ratio; CI—Confidence Interval.

## Data Availability

The raw data supporting the conclusions of this article will be made available by the authors on request.
